# Instantaneous Signal Loss simulation (InSiL) - an alternative algorithm for myocardial T1 mapping using the MOLLI sequence

**DOI:** 10.1186/1532-429X-16-S1-P26

**Published:** 2014-01-16

**Authors:** Jiaxin Shao, Yutaka Natsuaki, Bruce S Spottiswoode, Peng Hu

**Affiliations:** 1 Department of Radiological Sciences, David Geffen School of Medicine, University of California, Los Angeles, California, USA; 2 Siemens Healthcare, Malvern, Pennsylvania, USA; 3 Biomedical Physics Inter-Departmental Graduate Program, University of California, Los Angeles, California, USA

## Background

Myocardial T1 mapping is an emerging technique that is valuable for assessment of diffuse myocardial scar. Currently, the modified Look-Locker inversion-recovery (MOLLI) sequence has been widely used for T1 mapping and it uses 3-parameter exponential fitting with Lock-locker correction for T1 estimation. MOLLI is known to underestimate myocardial T1 values at longer T1s and/or at higher heart-rate. We propose an alternative T1 estimation method, Instantaneous Signal Loss simulation (InSiL), to improve T1 estimation accuracy and reduce the dependency of T1 estimation on heart rate for the MOLLI sequence.

## Methods

**Figure 1:**
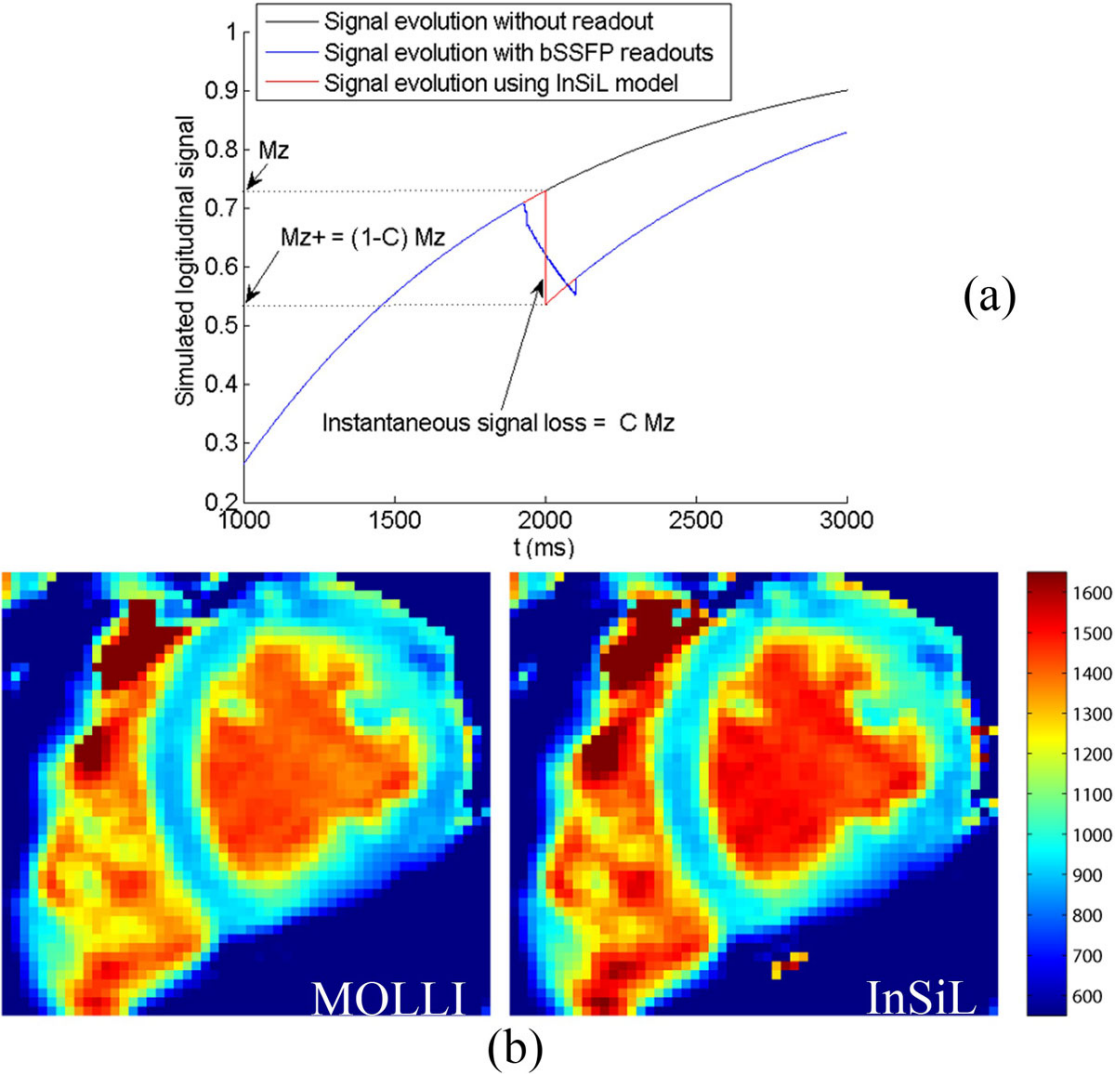
**(a) illustration of the InSiL model**. The longitudinal signal perturbation by each single-shot bSSFP imaging is simulated as instantaneous signal loss parameterized by factor C. (b) The short axis slice T1 maps of normal myocardium by the standard MOLLI approach and by InSiL at 1.5T, and the heart rate of this volunteer was 69 bpm. The average myocardial T1 based on InSiL was 20 ms higher than MOLLI (934 ± 28 ms vs. 914 ± 25 ms).

InSiL simulates the signal evolution of the MOLLI sequence for T1 calculation. The effect of longitudinal signal perturbation due to each single-shot imaging is parameterized as an instantaneous signal loss in longitudinal magnetization during the central k-space line by an unknown factor of C (0 ≤ C≤ 1), as illustrated in Figure 1a. Therefore, parameters M0, T1, and C can be solved so that the simulated signal matches best with the measured signal by the Levenberg-Marquardt algorithm. The standard MOLLI sequence with 3 inversion sets of 3, 3 and 5 images was studied on a 1.5T MR scanner. The T1 estimation accuracy over different T1s and heart rates (HR) by InSiL was evaluated against the standard MOLLI approach based on phantom data. Reference T1 values of phantoms were determined by spin-echo experiments. Non-contrast MOLLI image sets were acquired in ten healthy volunteers and the post-contrast MOLLI image sets were acquired 5 min, 10 min, and 15 min after contrast injection on two volunteers. Both InSiL and the standard MOLLI were used to calculate In vivo T1 values for comparison.

## Results

**Figure 2:**
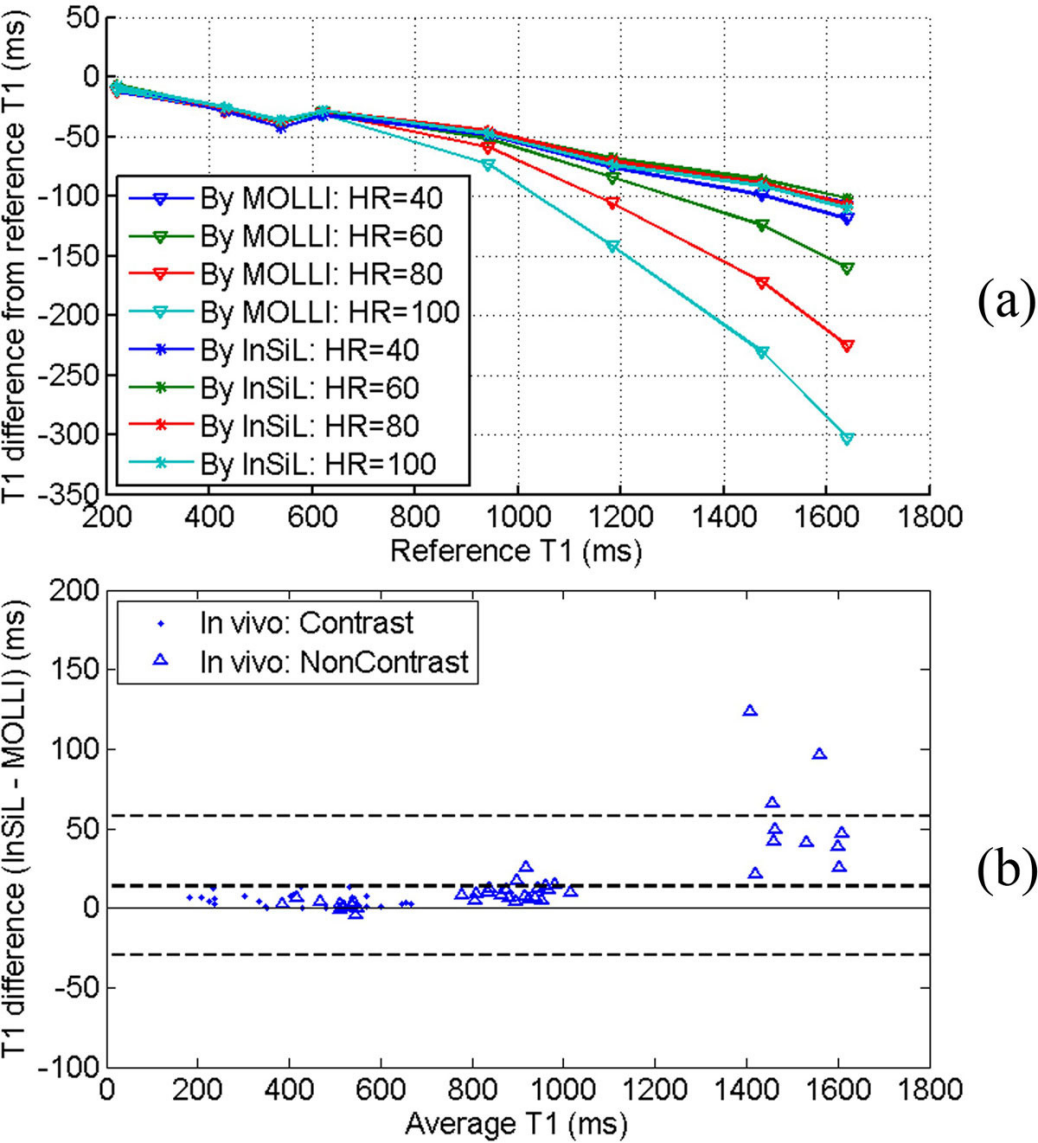
**(a) shows the T1 estimation error at different heart-rates by both methods in phantom studies**. Compared with the standard MOLLI approach, InSiL shows better T1 estimation accuracy and is less dependent on heart-rate. (b) shows the Bland-Altman plot of both methods in selected ROIs of myocardial, skeletal muscle, liver and blood at In vivo. InSiL produces larger T1 values compared with the MOLLI, especially for longer T1s, which correlates well with phantom results.

In phantom studies, both the standard MOLLI approach and InSiL approach underestimate T1 values compared to the reference. However, the MOLLI T1 values were 25.3 ms smaller than InSil T1 values on average for all phantom studies, and at heart rates ≥ 80 bpm, T1s >1000 ms, InSiL reduced MOLLI T1 underestimation by 104 ms on average (Figure 2a). Bland-Altman plot of in vivo T1 values using both methods (Figure 2b) shows that InSiL produces 14 ms larger T1 values compared with the MOLLI on average, and the difference is larger at longer T1s, which is the same trend as phantom results. Figure 1b shows an example of In vivo T1 maps by the standard MOLLI and by InSiL, where the average myocardial T1 based on InSiL was 20 ms higher than MOLLI (934 ± 28 ms vs. 914 ± 25 ms).

## Conclusions

Compared with the standard MOLLI, the proposed InSiL approach can reduce the heart-rate dependence in T1 estimation and achieves better T1 estimation accuracy, without any change in the pulse sequence.

## Funding

No funding.

